# In This Issue: Volume 117, Issue 5, May 2026

**DOI:** 10.1111/cas.70395

**Published:** 2026-05-01

## Expression of Glycoprotein B (gB) Correlates With Poor Prognosis in Nasopharyngeal Carcinoma

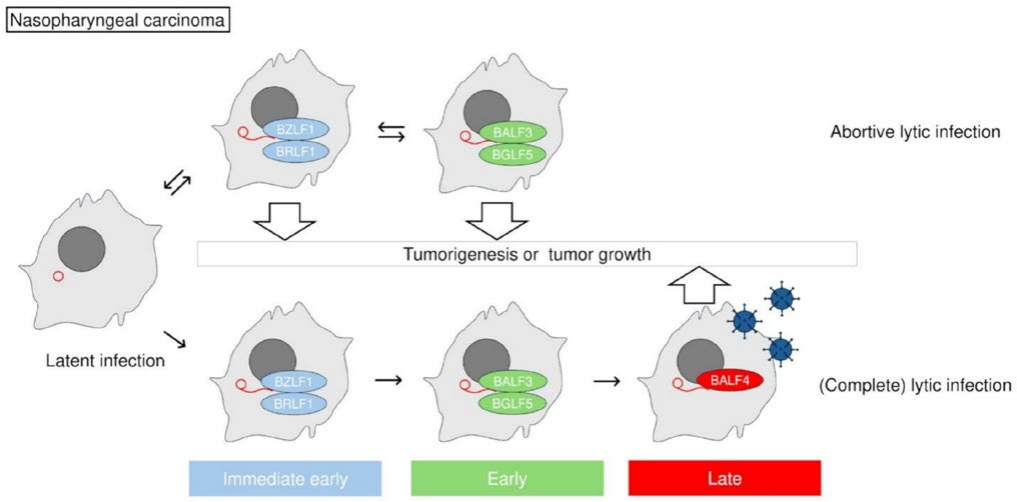

Epstein–Barr virus (EBV) is a common virus linked to several cancers, including nasopharyngeal cancer. It can exist in two states inside the body: a quiet phase and an active phase where new virus particles are produced. While the quiet phase has long been associated with cancer, the role of the active phase has remained unclear. Understanding how this virus behaves within cancer cells may provide important clues about disease progression and patient outcomes.

This study explored whether active viral infection occurs in nasopharyngeal cancer and how it affects prognosis. Using advanced imaging techniques, researchers were able to directly observe virus‐like particles in both cancer cells and patient tumor samples. They further monitored the release of these particles in real time and demonstrated that they were capable of infecting other cells, confirming ongoing viral activity. Importantly, they focused on a viral protein called glycoprotein B (gB), which is produced during active viral replication. Patients whose tumors showed higher levels of gB had a greater risk of disease progression, indicating a strong link between viral activity and worse outcomes.

Together, these findings show that active EBV infection, including the production and release of viral particles, plays a significant and previously underappreciated role in nasopharyngeal cancer. These results highlight the potential of monitoring viral activity as a biomarker for disease severity and progression. Detecting proteins like gB could help identify high‐risk patients and support the development of future treatments aimed at targeting viral activity.

https://onlinelibrary.wiley.com/doi/full/10.1111/cas.70341

## Circ‐0030167/IGF2BP1 Induces Mitophagy‐Mediated Ferroptosis via HMOX1 mRNA Stabilization in Pancreatic Cancer

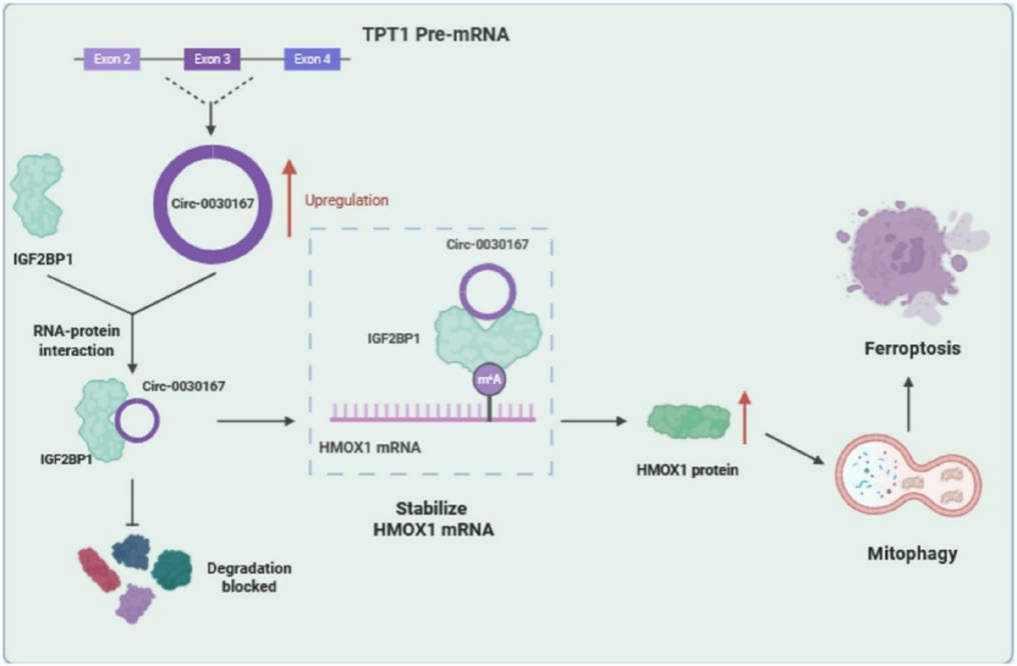

Pancreatic cancer (PC) is one of the deadliest forms of cancer with a low patient survival rate. However, the available treatment strategies are inadequate, necessitating identification of mechanisms that play a role in tumor growth and can be targeted for treatment of PC.

Recently, circular ribonucleic acids (circRNAs), a type of single‐stranded RNA that forms a closed loop, have been reported to regulate tumor growth and spread in PC. While targeting the regulatory mechanism may be a novel treatment strategy, the underlying mechanism in PC remains unknown.

To elucidate this, researchers employed a circRNA called circ‐0030167 and investigated whether it exerts regulatory function by pushing cancer cells toward mitophagy, a process in which cells break down their own damaged mitochondria (an organelle that generates energy for cellular function), and ferroptosis, an iron‐dependent form of cell death.

The researchers found that circ‐0030167 was present at lower levels in PC tissue compared to normal pancreatic tissue, and higher levels of it were linked to better survival. In addition, they observed that high levels of circ‐0030167 triggered mitophagy and ferroptosis, indicating a significant protective effect of circ‐0030167 as damaged mitochondria can fuel tumor growth.

Moreover, they mapped out the mechanistic pathway and noted that circ‐0030167 interacts and stabilizes the IGF2BP1 protein, which further stabilizes heme oxygenase‐1 (HMOX1) mRNA. HMOX1 protein is involved in stress responses and promotes mitophagy and ferroptosis. Furthermore, experiments in mice models revealed that high levels of circ‐0030167 reduced tumor growth in PC.

Together, these findings suggest that circ‐0030167 may suppress tumor growth in PC through activation of mitophagy and ferroptosis by interacting with IGF2BP1 and stabilizing HMOX1 mRNA. Thus, targeting this novel mechanism may be a promising strategy for treatment of PC.

https://onlinelibrary.wiley.com/doi/full/10.1111/cas.70356

## Mitochondrial Transfer in the Tumor Microenvironment

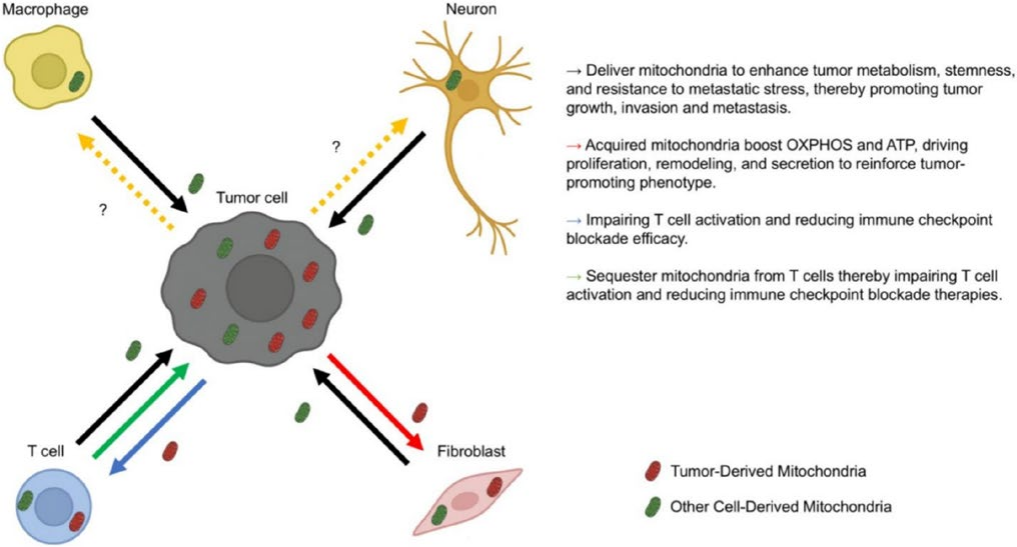

Mitochondria are widely known as the “powerhouses” of the cell because they generate energy in the form of ATP. However, they also play essential roles in regulating cell growth, survival, apoptosis, and responses to stress. Unlike most cellular components, mitochondria contain their own DNA, reflecting their origin from ancient bacteria that became integrated into early cells. This unique feature allows them to remain highly adaptable and responsive to changing cellular conditions.

In this review, Omae et al. describe mitochondrial transfer, a process in which mitochondria move from one cell to another. This phenomenon is increasingly recognized as biologically important, particularly in cancer. Within the tumor microenvironment, cells experience stressful conditions such as low oxygen levels and inflammation. These conditions can trigger mitochondrial transfer through several mechanisms, including thin tube‐like connections between cells, extracellular vesicles, and direct cell‐to‐cell contact.

Such transfers can significantly influence cancer progression. Neighboring supportive cells can donate mitochondria to tumor cells, helping them maintain energy production and survive in hostile environments. This added support may also enable tumor cells to resist therapeutic treatments. At the same time, tumor cells can transfer mitochondria to immune cells, such as T cells, which can impair their ability to recognize and attack cancer effectively.

These findings suggest that mitochondrial transfer plays a dual role in promoting tumor survival and weakening the body's natural immune defenses. As a result, it represents a promising target for future therapies. Approaches that block harmful mitochondrial transfer to tumor cells or restore immune function may improve treatment outcomes. However, further research is needed to develop safe and precise methods to control this process in clinical settings.

https://onlinelibrary.wiley.com/doi/full/10.1111/cas.70342

